# Monitoring myocardial oxygenation response to respiratory maneuvers using real-time magnetic resonance imaging

**DOI:** 10.1016/j.jocmr.2026.102751

**Published:** 2026-06-03

**Authors:** Nora Vogt, Sonia Lebza, Jacques Felblinger, Gilles Bosser, Freddy Odille

**Affiliations:** a IADI U1254, Inserm, Université de Lorraine, Nancy, France; b CIC-IT 1433, Inserm, Université de Lorraine, CHRU de Nancy, Nancy, France; c Service de Cardiologie Congénitale et Pédiatrique, CHRU de Nancy, Nancy, France

**Keywords:** Breathing maneuvers, Microvascular function, Oxygenation-sensitive cardiovascular magnetic resonance imaging, Real-time MRI

## Abstract

**Background:**

Oxygenation-sensitive (OS) cardiovascular magnetic resonance (CMR), using breathing maneuver-induced stress and blood oxygen level-dependent contrast, is a well-tolerated, non-invasive approach for assessing myocardial oxygenation. This study evaluated two real-time CMR sequences and a semi-automated analysis workflow to enable continuous, motion-robust assessment of myocardial oxygenation dynamics, aiming to overcome limitations of electrocardiogram (ECG)-triggered acquisitions and reduce manual evaluation requirements.

**Methods:**

Signal intensity dynamics of ten young healthy female volunteers were analyzed from images acquired on a 3T scanner using an ECG-triggered balanced steady-state free precession (bSSFP) sequence, a real-time (RT) bSSFP sequence, and a RT fast low-angle short (FLASH) sequence. Images were obtained in a mid-ventricular slice while participants performed a breathing maneuver consisting of normal breathing, paced hyperventilation, and a prolonged breath hold. A semi-automated processing pipeline was implemented to generate motion-resolved oxygenation maps using retrospective cardiac phase binning, deformable image registration, and automated segmentation.

**Results:**

The RT sequences captured end-systolic global myocardial signal intensity change (Δ*SI*, relative to reference frames at breath-hold start) with dynamics comparable to those of the ECG-triggered bSSFP sequence. As expected in healthy volunteers, Δ*SI* decreased during hyperventilation and recovered during the prolonged breath-hold. At 20–30 s, oxygenation responses at end-systole were 3.95%[3.36, 7.50] for ECG-triggered bSSFP, 5.47%[3.31, 9.08] for RT bSSFP, and 3.24%[1.99, 4.80] for RT FLASH. RT FLASH showed fewer artifacts but lower contrast and smaller Δ*SI* than the bSSFP sequences, with inter-sequence differences appearing modest in mixed-effects analysis.

**Conclusions:**

This exploratory study demonstrates the feasibility of combining real-time MR sequences with a semi-automated processing pipeline for continuous assessment of *T*2∕*T*2*-related myocardial oxygenation dynamics. Potential confounders, including through-plane motion and physiological drift, warrant further investigation.

## Graphical abstract

**Figure:**
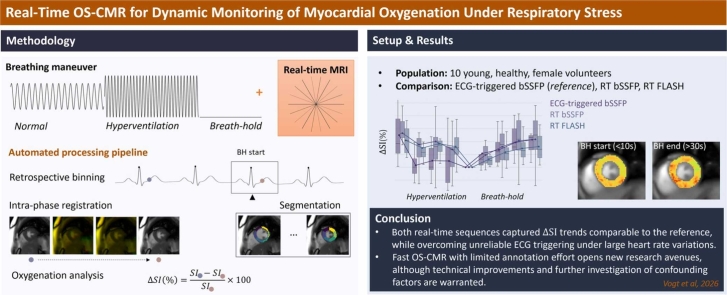

## 1 Introduction

Many patients with suspected myocardial ischemia undergo invasive, ionizing angiography to evaluate coronary anatomy and physiology, yet often show no obstructive disease. This emphasizes the need for non-invasive methods to assess coronary and microvascular function. Stress cardiovascular magnetic resonance imaging (CMR) evaluates myocardial perfusion but requires contrast agents and pharmacologic stress, whereas oxygenation-sensitive (OS) CMR provides complementary information by assessing myocardial oxygenation through alterations in *T*2 and *T*2* MR signals that reflect the hemoglobin’s oxygen (*O*_2_) saturation. In this work, participants followed a stress-inducing breathing maneuver (BM) as proposed by Fisher et al. [1], [2], a method demonstrated to provoke myocardial responses similar to pharmacologic vasodilation with adenosine, but with improved safety and patient comfort [1]. This maneuver involves a minute of paced hyperventilation followed by a subsequent prolonged breath-hold. Hyperventilation is considered to induce vasoconstriction with reduced blood flow and myocardial *T*2∕*T*2*, whereas the subsequent breath-hold is thought to promote vasodilation, increasing blood flow and myocardial *T*2∕*T*2* as oxygen supply progressively exceeds myocardial *O*_2_ demand. The oxygenation response is typically quantified as the ratio of myocardial signal intensity at the end (∼30 s) versus the start of the breath-hold. The feasibility of OS-CMR using breathing maneuvers and a *T*2-prepared balanced steady-state free precession (bSSFP) sequence has been demonstrated in multiple studies involving healthy volunteers [1], [3], [4], [5], [6], patients [3], [4], [5], [6], and swine [7]. As relying on ECG-triggered imaging, these sequences are susceptible to R-peak detection errors as well as inconsistent acquisition window placement caused by stress-induced heart rate variability. Yielding a measurement every fourth heartbeat, these sequences are furthermore limited in temporal resolution. To enable continuous imaging of the full cardiac cycle during breathing maneuvers, Guensch et al. [8] recently presented an oxygenation-sensitive real-time sequence with a temporal resolution of 49.8 ms. Building on this concept, this study investigated fast low-angle shot (FLASH) and bSSFP real-time CMR sequences with an interleaved radial spoke arrangement [9] and temporal resolutions of 17.5–30 ms for oxygenation-sensitive imaging, while introducing a semi-automated processing pipeline to generate motion-resolved oxygenation maps, incorporating retrospective cardiac phase binning, deformable respiratory motion compensation, and automated cardiac segmentation.

## 2 Methods

### 2.1 Data acquisition

Ten healthy female participants were recruited as part of the ethics committee-approved EDEN study (ClinicalTrials.gov identifier: NCT05218460). They were scanned on a 3T (Magnetom Prisma, Siemens Healthineers, Erlangen, Germany) scanner while performing a video-guided breathing maneuver comprising 60 s of normal breathing (*Normal*), 60 s of paced hyperventilation (30 breaths/min) (HV), and a 30–40 s breath-hold at end-expiration (BH). Repeating this maneuver, mid-ventricular short-axis acquisitions were obtained using three sequences: ECG-triggered bSSFP with measurements every fourth heartbeat at end-systole; real-time bSSFP with a temporal resolution of 30 ms [9]; and real-time FLASH with a temporal resolution of 17.5 ms [9]. Detailed sequence parameters are provided in the Supplemental Material. Subjects were monitored using a four-lead ECG sensor (Siemens Healthineers) and a respiration belt (Maglife, Schiller Médical, Wissembourg, France).

### 2.2 Processing framework

As detailed in the Supplemental Material, the proposed semi-automated processing framework comprised retrospective binning of reconstructed frames into 20 cardiac phases, deformable registration of all frames to cardiac-phase-matched breath-hold start reference frames, and nnU-Net-based segmentation of the myocardium and ventricles. Quality control involved manual contour correction, artifact delineation, and exclusion of frames with incorrect cardiac-phase assignment, pronounced through-plane motion, or poor image quality. Deep inhalation samples, associated with the largest through-plane motion, were excluded based on a respiration signal amplitude cutoff (see Supplemental Methods). The reviewed myocardial mask was divided into six American Heart Association (AHA) segments, and global and segment-wise average signal intensities were computed, excluding artifacted pixels. Signal intensity changes (Δ*SI*) were calculated relative to the breath-hold start references. For the segment-wise analysis, segments with more than 33% artifacted pixels were excluded from the cohort statistics.

### 2.3 Statistical analysis

The primary endpoint was the end-systolic myocardial oxygenation response at breath-hold end (global Δ*SI* at 20–30 s). Measurements were averaged for 10-second intervals to account for differences in sampling rates across sequences. We further assessed global and segment-wise Δ*SI* changes throughout the entire breathing maneuver and across the cardiac cycle. A linear mixed-effects model was fitted using restricted maximum likelihood (REML) to analyze Δ*SI*, with sequence type, breathing interval, their interaction, and RR interval (ECG R-to-R interval, a proxy for heart rate) included as fixed effects, and subject-specific random intercepts to account for repeated measures within subjects. Post-hoc pairwise comparisons were derived from model-based marginal contrasts to assess differences between breathing intervals within each sequence and between sequence types within each breathing interval. Additional statistical assessments for sequence comparisons, hyperparameter sensitivity analyses, and repeatability are provided in the Supplemental Material. No formal adjustment for multiple comparisons was performed. Accordingly, p-values are provided for descriptive purposes, with interpretation based primarily on estimated effect sizes and their associated confidence intervals. Statistical analyses were performed in Python (v3.10.14) using statsmodels (v0.14.5) and marginaleffects (v0.0.12).

## 3 Results

**Fig. 1:**
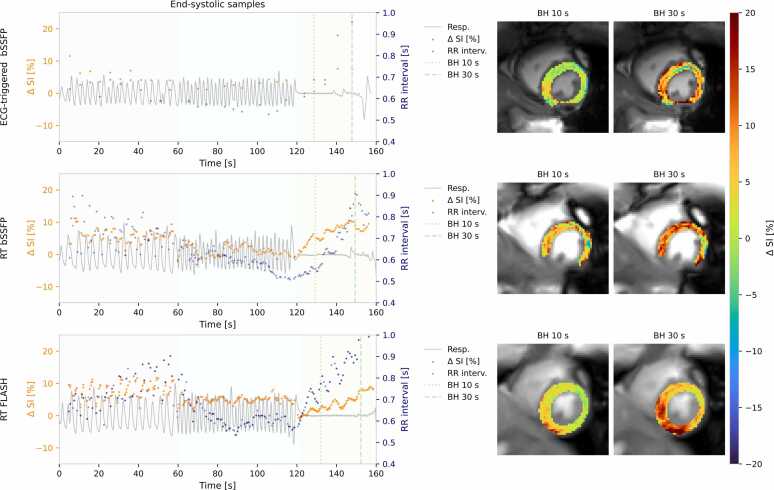
Global signal intensity change (Δ*SI*, relative to the breath-hold start reference frame) time profiles and example oxygenation maps for subject 5 with comparably high global Δ*SI*. Left: Global Δ*SI* and RR interval throughout the breathing maneuver (gray = normal breathing, blue = hyperventilation, green = breath-hold) for, from top to bottom, ECG-triggered bSSFP, real-time bSSFP, and real-time FLASH. Y-axis labels and ticks are color-coded to match the respective data series (orange = Δ*SI*, blue = RR interval). Right: Oxygenation maps at two breath-hold time points (samples closest to 10 s and 30 s of breath-hold), corresponding to the vertical dashed lines on the left. RR interval refers to the ECG R-to-R interval, a proxy for heart rate. *ECG* electrocardiogram, *bSSFP* balanced steady-state free precession, *FLASH* fast low-angle shot, *HV* hyperventilation, *BH* breath hold, *RT* real time

**Fig. 2:**
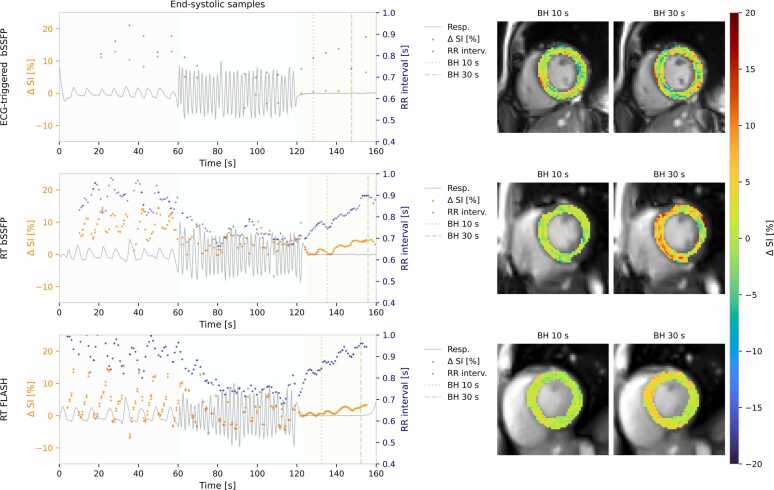
Global signal intensity change (Δ*SI*, relative to the breath-hold start reference frame) time profiles and example oxygenation maps for subject 3 with medium global Δ*SI*. Left: Global Δ*SI* and RR interval throughout the breathing maneuver (gray = normal breathing, blue = hyperventilation, green = breath-hold) for, from top to bottom, ECG-triggered bSSFP, real-time bSSFP, and real-time FLASH. Y-axis labels and ticks are color-coded to match the respective data series (orange = Δ*SI*, blue = RR interval). Right: Oxygenation maps at two breath-hold time points (samples closest to 10 s and 30 s of breath-hold), corresponding to the vertical dashed lines on the left. RR interval refers to the ECG R-to-R interval, a proxy for heart rate. *ECG* electrocardiogram, *bSSFP* balanced steady-state free precession, *FLASH* fast low-angle shot, *HV* hyperventilation, *BH* breath hold, *RT* real time

The ten participants (median age 20.5 years; IQR 19–25.8) followed the breathing instructions without discomfort and performed breath-holds of 39.7 [37.4, 40.9] seconds. Table 1 and Suppl. Fig. 3 show end-systolic Δ*SI* evolution patterns consistent with expected vasoconstriction-related reductions in oxygen delivery and subsequent vasodilation with relative oxygen oversupply—namely, a decrease during hyperventilation followed by recovery during the subsequent breath-hold. However, as data were acquired continuously throughout the maneuver, measurements during deep breathing may have been biased by through-plane motion related to respiration. In addition, imaging at end-systole could have introduced further bias due to cardiac motion-related through-plane displacement. Effect analysis (Suppl. Table 2), adjusting for RR interval as a fixed effect, indicated that estimated end-systolic oxygenation responses (Δ*SI*, %) increased with breath-hold duration relative to breath-hold start, with estimated changes in Δ*SI* of 2.39% for BH <10 s (95% CI: −0.55–5.33, p = 0.11), 3.35% for BH 10–20 s (0.45–6.26, p = 0.02), 4.89% for BH 20–30 s (1.80–7.98, p<0.01), and 5.12% for BH >30 s (1.86–8.37, p<0.01). Sequence type, RR interval, and most sequence-breathing interval interactions showed little evidence of an association with Δ*SI*. Random subject-level variability was 2.52. Intra-sequence comparisons across breathing intervals suggested increasing Δ*SI* throughout the maneuver for all three sequences. The ECG-triggered sequence exhibited the largest signal intensity change between normal breathing and hyperventilation (Suppl. Table 3). As the acquisition order was fixed and temporal drift may have contributed to the observed Δ*SI* changes, inter-sequence differences should be interpreted cautiously. The strongest disagreement was observed for deep breathing intervals (Suppl. Table 4). Example oxygenation response maps and Δ*SI* profiles, comparing the ECG-triggered bSSFP, RT bSSFP, and RT FLASH sequences, are shown in Figs. 1-2. Detailed statistical analyses, investigating inter-subject, inter-sequence, and inter-phase variations, as well as analysis sensitivity and repeatability, are provided in the Suppl. Materials.

**Table 1:** Global and septal end-systolic Δ*SI* changes, computed relative to reference frames at breath-hold start, across breathing maneuvers

Sequence	BM group	Samples (#)	Global Δ*SI* (%)	Septal Δ*SI* (%)	RR interval (s)
ECG-triggered bSSFP	Normal	10	4.59 [2.71, 9.40]	3.50 [2.79, 7.32]	0.78 [0.70, 0.84]
	HV 30–40 s	9	0.02 [−1.22, 1.66]	−0.07 [−1.76, 0.42]	0.61 [0.57, 0.68]
	HV 40–50 s	9	0.04 [−0.46, 3.73]	−0.62 [−1.21, 5.30]	0.64 [0.59, 0.72]
	HV 50–60 s	8	1.22 [−2.99, 3.99]	0.39 [−2.91, 4.92]	0.66 [0.63, 0.73]
	BH <10 s	9	1.57 [0.41, 2.49]	1.48 [1.10, 3.06]	0.75 [0.69, 0.79]
	BH 10–20 s	10	3.02 [0.98, 4.86]	3.21 [2.09, 5.97]	0.80 [0.70, 0.86]
	BH 20–30 s	9	3.95 [3.36, 7.50]	3.32 [1.79, 7.77]	0.82 [0.79, 0.93]
	BH >30 s	7	5.79 [3.38, 6.18]	4.22 [2.31, 5.63]	0.81 [0.78, 0.86]
RT bSSFP	Normal	10	6.52 [5.75, 8.91]	7.67 [5.87, 10.91]	0.82 [0.78, 0.87]
	HV 30–40 s	10	4.46 [1.97, 5.61]	3.82 [1.95, 6.06]	0.64 [0.60, 0.75]
	HV 40–50 s	10	3.99 [1.55, 5.84]	3.31 [1.01, 6.16]	0.68 [0.62, 0.74]
	HV 50–60 s	10	3.47 [0.10, 3.90]	2.21 [−0.28, 4.85]	0.69 [0.61, 0.74]
	BH <10 s	10	2.51 [0.79, 4.07]	2.73 [1.19, 5.63]	0.76 [0.63, 0.84]
	BH 10–20 s	10	4.40 [1.43, 6.50]	4.20 [0.73, 7.93]	0.80 [0.66, 0.85]
	BH 20–30 s	10	5.47 [3.31, 9.08]	6.56 [2.14, 10.37]	0.83 [0.76, 0.88]
	BH >30 s	10	6.27 [3.44, 9.04]	6.28 [2.96, 11.95]	0.86 [0.81, 0.92]
RT FLASH	Normal	10	5.21 [2.80, 7.50]	5.95 [4.67, 8.54]	0.82 [0.77, 0.88]
	HV 30–40 s	10	2.91 [2.01, 3.83]	4.11 [3.59, 4.59]	0.73 [0.67, 0.74]
	HV 40–50 s	10	4.30 [3.05, 5.14]	4.94 [4.26, 5.70]	0.74 [0.68, 0.76]
	HV 50–60 s	10	3.79 [2.52, 5.77]	4.00 [3.13, 6.91]	0.73 [0.66, 0.78]
	BH <10 s	10	1.27 [0.46, 2.18]	1.50 [1.07, 2.29]	0.82 [0.68, 0.87]
	BH 10–20 s	10	2.66 [1.39, 3.82]	3.39 [2.48, 5.37]	0.86 [0.76, 0.89]
	BH 20–30 s	10	3.24 [1.99, 4.80]	4.35 [3.27, 5.85]	0.88 [0.81, 0.94]
	BH >30 s	9	3.51 [1.85, 6.05]	4.49 [3.76, 7.17]	0.95 [0.84, 0.98]

Data are expressed as median [IQR].

*RR interval* ECG R-to-R interval, *BM* breathing maneuver, *SI* signal intensity, *ECG* electrocardiogram, *bSSFP* balanced steady-state free precession, *FLASH* fast low-angle shot, *HV* hyperventilation, *BH* breath hold, *RT* real time

## 4 Discussion

Real-time sequences have previously shown promise for continuous OS-CMR under respiratory motion [8]. The present work compared two radially undersampled real-time sequences, RT bSSFP (*T*2 contrast) and RT FLASH (*T*2* contrast), and introduced a semi-automated pipeline that limits manual contouring to reference breath-hold frames and aligns image frames of each cardiac phase via deformable registration. As discussed in the Supplemental Material, user intervention was nevertheless required to review R-peak detections, to correct the automated segmentation masks (with most interventions required for the RT FLASH), to delineate artifacted regions, and to perform quality checks. Technical improvements thus include advanced automated R-peak detection, FLASH contrast-specific segmentation model fine-tuning, and automated outlier-frame identification. Following [4], [6], our statistical analysis of oxygenation response focused on end-systolic frames during the breath-hold period. Further work is required to interpret the pronounced Δ*SI* variations observed across the cardiac cycle, as diastolic phases may be more sensitive to segmentation and registration errors, yet less affected by through-plane motion due to cardiac contraction. Dresselaers et al. [10] showed that through-plane motion velocity and spin magnetization history strongly influence signal intensity in 2D bSSFP cine imaging, producing cyclic SI variations throughout the cardiac cycle. These findings highlight the limitations of 2D acquisitions, emphasize the importance of accurate cardiac synchronization, and suggest caution when quantitatively evaluating signal intensity under motion. RT FLASH produced the smoothest oxygenation maps, although further optimization of T2*-weighting is warranted (quantitative heterogeneity and ablation studies are provided in the Supplemental Materials). The bSSFP sequences provided higher contrast but were susceptible to *B*_0_-related banding; for RT bSSFP, radial undersampling occasionally introduced streaking artifacts, which, in addition to through-plane motion and minor cardiac phase mismatches, may have contributed to the observed heterogeneity of the derived oxygenation maps. Inter-sequence comparisons may have been further influenced by differences in artifact distributions and frame exclusion rates, summarized in Suppl. Table 6. Further studies should investigate how population characteristics (e.g., age, gender) and sequence parameters affect the oxygenation response, as our measured Δ*SI* values—averaged across 10-second intervals—were lower than those reported in prior work (e.g., Hillier et al. [5], [6], Fischer et al. [1], [3], [4]). This exploratory study included only female volunteers to align with the anticipated clinical application in patients with ischemia with non-obstructive coronary arteries (INOCA), a condition that predominantly affects women. Additional limitations include the fixed sequence order, which may have influenced the physiological response and prevented causal attribution of differences between sequences. Although the RR interval was included as a fixed effect to account for heart rate-related differences, other sequence order effects, such as time-on-task, physiological drift, or scanner-related drift, remain potential confounders in this study’s exploratory inter-sequence comparisons. A single-subject same-day repeatability assessment in a single subject indicated good repeatability of global end-systolic Δ*SI* for RT FLASH across breathing intervals (ICC = 0.90, 95% CI: 0.52–0.98), but confirmation in a larger sample is needed. The lack of physiological monitoring (*O*_2_, *CO*_2_, blood pressure) limits interpretability and should be addressed in future studies. Improved breathing instructions are needed to ensure consistent hyperventilation stress and accurate breath-hold timing. As single slices offer limited diagnostic value, solutions for expanded cardiac coverage are required. Ultimately, evaluation in patients with perfusion abnormalities is essential to determine clinical applicability.

## 5 Conclusion

We explored the potential of real-time sequences and semi-automated processing pipelines for monitoring signal-intensity changes during breathing maneuvers in a small cohort of young female participants. While this approach offers potential for studying temporal dynamics, further investigations are needed to limit confounders, reduce measurement variability, and validate the findings in larger study populations. Important research directions include the development of reconstruction techniques that enable whole-heart coverage, as well as technical improvements to reduce manual intervention and enhance robustness.

## Funding

This research was funded by the ANR grant SWEETHEART (ANR-23-CE19–0030) from the national call on “Technologies pour la santé”. The project was co-funded by the French State-Region contract CPER 2015–2020 (Contrat de Plan Etat Région- IT2MP Innovations Technologiques, Modélisation et Médecine Personnalisée) and by the European Union through the European Regional Development Fund “FEDER-FSE Lorraine et Massif des Vosges 2014–2020.”

## Author contributions

**Nora Vogt:** Writing – original draft, Visualization, Validation, Software, Methodology, Data curation, Conceptualization. **Sonia Lebza:** Writing – review & editing, Visualization, Validation, Methodology, Data curation. **Jacques Felblinger:** Writing – review & editing, Data curation, Conceptualization. **Gilles Bosser:** Writing – review & editing, Conceptualization. **Freddy Odille:** Writing – review & editing, Supervision, Project administration, Methodology, Funding acquisition, Data curation, Conceptualization.

## Ethics approval and consent

This study was approved by an ethics committee and informed written consent was obtained (ClinicalTrials.gov identifier: NCT05218460).

## Consent for publication

The source code will be available upon reasonable request for academic research purposes. Acquired data of participants will not be openly available due to reasons of sensitivity. Data are located in controlled access data storage at the University Hospital in Nancy.

## Declaration of generative AI in scientific writing

During the preparation of this work, the first author used GPT-4 and GPT-5 to improve the grammar and readability of certain paragraphs. After using this tool, the author reviewed and edited the content as needed and takes full responsibility for the content of the publication.

## Declaration of competing interests

The authors declare no conflicts of interest.

## Appendix A Supplementary material

Supplementary materialSupplementary materialSupplementary material
