# Supplementary material for: Monitoring myocardial oxygenation response to respiratory maneuvers using real-time magnetic resonance imaging
Source: J Cardiovasc Magn Reson. 2026 Jun 3;28(2):102751. doi: 10.1016/j.jocmr.2026.102751 (PMC13288510; doi:10.1016/j.jocmr.2026.102751)
Supplement: Supplementary file 1 — Supplementary materialSupplementary materialSupplementary material [file mmc1.pdf]

# Contents


1 Supplemental Methods 1
1.1 Data Acquisition . . . 1
1.2 Processing Framework . . . 1

2 Supplemental Results 3
2.1 Data Acquisition . . . 3
2.2 Statistical Analysis . . . 3
2.2.1 Linear Mixed-Effect Analysis . . . 3
2.2.2 Intra- and Inter-sequence Comparisons . . . 3
2.3 AHA Segment Analysis . . . 4
2.4 Cardiac Cycle Analysis . . . 5
2.5 Sensitivity Analysis . . . 6
2.6 Repeatability Analysis . . . 7
2.6.1 Quality Control Statistics . . . 7
2.6.2 Repeated Reading . . . 8
2.6.3 Repeated Acquisition . . . 9
2.7 Echo Time Ablations . . . 9


# 1. Supplemental Methods

## 1.1. Data Acquisition

Mid-ventricular short-axis (SAx) images were acquired for the following three sequences, using a fixed sequence order:

- **ECG-triggered bSSFP:** spatial resolution $2 \times 2 \times 10$ mm, measurement every fourth heartbeat, TR/TE: 295 ms/1.51 ms, echo spacing: 3.01 ms, flip angle: 35°, bandwidth: 1302 Hz, FOV: 312×384 mm, $T2$ preparation, GRAPPA acceleration factor 3. The trigger delay was adjusted for each volunteer using a breath-hold cine pre-scan to yield end-systolic frames.
- **Real-time bSSFP:** spatial resolution $1.92 \times 1.92 \times 10$ mm, temporal resolution 30 ms, TR/TE: 3.7 ms/1.85 ms, flip angle: 35°, bandwidth: 758 Hz, FOV: 383 × 383 mm. NLInv reconstruction [1, 2]: 9 spokes per frame, 5 turns, temporal median filtering over 5 adjacent image frames, 2 preparation scans.
- **Real-time FLASH:** spatial resolution 1.92×1.92×10 mm, temporal resolution 17.5 ms, TR/TE: 2.33 ms/1.61 ms, flip angle: 5°, bandwidth: 1667 Hz, FOV: 383 × 383 mm. NLInv reconstruction [1, 2]: 9 spokes per frame, 5 turns, temporal median filtering over 5 adjacent image frames, 2 preparation scans.

The ECG was captured using a four-lead Siemens sensor at 0.4 kHz and respiration was monitored using a respiration belt at 1 kHz (Maglife, Schiller Médical, Wissembourg, France). A signal analyzer and event controller [3] was used to synchronize these recordings with the MRI data.

## 1.2. Processing Framework

The proposed processing pipeline is visualized in Fig.1. Respiration signals were analyzed to identify normal (N), hyperventilation (HV), and breath-hold (BH) periods. Retrospective cardiac binning into 20 cardiac bins was performed by first reviewing and correcting scanner-provided R-peak annotations against the recorded ECG using a Python interface, and then discretizing cardiac phases obtained from the model of Feinstein et al. [4], distinguishing systolic ($(t - t_{QRS}) < \Delta_{sys}$) and diastolic frames $t$:

$$c_{systole}(t) = \frac{t - t_{QRS}}{\Delta_{sys}} \frac{\overline{\Delta_{sys}}}{\overline{\Delta_{RR}}} \text{ and } c_{diastole}(t) = \frac{t - t_{QRS} - \Delta_{sys}}{\Delta_{dia}} \frac{\overline{\Delta_{dia}}}{\overline{\Delta_{RR}}} + \frac{\overline{\Delta_{sys}}}{\overline{\Delta_{RR}}}, \quad (1)$$

with $\Delta_{RR}$ denoting the current RR-interval duration, $t_{QRS}$ the preceding QRS location, $\Delta_{sys}$ the systolic phase duration, $\Delta_{dia} = \Delta_{RR} - \Delta_{sys}$, $\Delta_{sys} = (546\,\text{ms} - 2.1 HR_t)/1000$, $HR_t = 60/\Delta t_{RR}$, and $\overline{\star}$ the full record mean values.

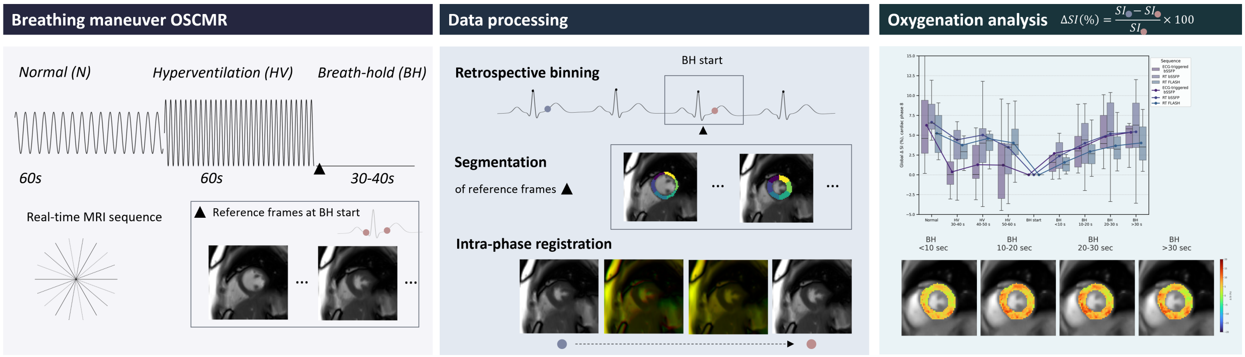


**Figure 1:** Pipeline overview: Volunteers were continuously imaged with a radial interleaved real-time sequence [1] during 60 s of normal breathing, 60 s of hyperventilation, and a prolonged breath-hold. After retrospective cardiac binning, all frames were aligned to a cardiac-phase–matched breath-hold start reference frame (one per cardiac phase) using deformable registration. Myocardial masks and AHA segments were generated from the reference frames and used to compute global and segment-wise myocardial oxygenation response ($\Delta SI$) metrics.

All frames from the prospectively triggered bSSFP sequence were assigned to a single cardiac bin ($c = 8$). For each cardiac bin, the sample closest to the breath-hold start was chosen as the reference frame. The left ventricle, right ventricle, and myocardium were segmented in these reference frames (20 for the real-time sequences, 1 for the ECG-triggered sequence) using a 2D nnU-Net model trained on the M&Ms Challenge dataset [5] (code version 1.7.1; 2D model (tta=True; mixed_precision=True; mode=normal; step_size=0.5; chk=model_final_checkpoint; disable_postprocessing=False). Image frames were cropped to the bounding box of the cardiac masks, adding a border of 10 pixels in all dimensions. After bin assignment, all frames were non-rigidly registered to the corresponding reference using the symmetric diffeomorphic registration (SyN) algorithm and a cross-correlation similarity metric from the ANTsPy/DIPY library [6] (code version 1.10.0; SymmetricDiffeomorphicRegistration algorithm using the CCMetric (dim=2, sigma_diff=2.0), radius=4; level_iters=[100, 100, 25]; step_length=0.25; ss_sigma_factor=0.2; opt_tol=1e-5; inv_iter=20; inv_tol=1e-3). Myocardial masks were divided into six regions approximating segments 7–12 of the mid-ventricular SAx plane in the 17-segment AHA model [7]. Automated segment definition was based on angular separation relative to the LV centroid, with septal segments 8 and 9 divided by the axis connecting the LV and RV centroids. Starting from this reference angle, the myocardium was segmented into six consecutive 60°sectors. Intermediate results were reviewed in a Python interface for quality control, including manual contour corrections and, where needed, manual delineation of regions unsuitable for analysis (i.e., banding artifacts and regions affected by registration failure). Registration failures were defined as regions where automated image alignment was unsuccessful throughout the breathing maneuver, typically due to warping of right ventricular tissue into the myocardial mask. Although registration failures do not represent imaging artifacts per se, both were jointly annotated and are hereafter referred to as artifacts. For the real-time sequences, the first 100 frames of the normal breathing maneuver were excluded to allow image stabilization, corresponding to approximately 3 s for the RT bSSFP sequence and 1.75 s for the RT FLASH sequence. Frames failing quality control—due to poor image quality, cardiac synchronization, or strong through-plane motion/registration errors—were excluded from further analysis. As the strongest through-plane motion was observed during deep inhalation, we excluded all samples exceeding the 80th percentile of the global pre-processed respiration recording amplitude from the analysis. Pre-processing involved a first-order Butterworth low-pass filter for denoising (cutoff 3 Hz) and a first-order Butterworth high-pass filter for drift correction (cutoff 0.1 Hz). Frames of the real-time sequences, grouped by breathing maneuver and cardiac phase, were then median filtered along the temporal dimension using a filter window of size 5. Due to the higher temporal resolution of real-time sequences compared with ECG-triggered sequences, median filtering was applied within each cardiac phase bin to smooth temporal variations while preserving anatomical details. In addition to oxygenation maps, which represent pixel-wise signal intensity changes between a selected frame and the corresponding BH start reference frame, we computed global and segment-wise oxygenation responses by first calculating the mean intensity per segment (or global myocardium, excluding artifacted pixels), and then computing the difference between (segment mean) signal

**Table 1**
Population characteristics (expressed as median [IQR] or frequency (%)).

| Characteristic | Cohort (n=10) |
|---|---|
| Age (years) | 20.5 [19, 25.8] |
| Sex (female) | 10 (100) |
| Weight (kg) | 58 [57, 68] |
| Height (cm) | 165 [164, 168] |

intensities of frame t ($SI_{\mathrm{t}}$) and reference frame ($SI_{\mathrm{ref}}$):

$$\Delta SI(t) = \begin{cases} \frac{SI_t - SI_{\mathrm{ref}}}{SI_{\mathrm{ref}}} \times 100, & \text{if } SI_{\mathrm{ref}} > 1e-6 \text{ and both } SI_{\mathrm{ref}}, SI_t \text{ are finite,} \\ \text{NaN (excluded)}, & \text{otherwise.} \end{cases}$$

For the AHA segment analysis, segments with more than 33% of artifacted pixels were excluded from the cohort statistics (Section 2.3). Examples of acquired image sequences, cardiac phase assignments, and intra-phase registration are provided as supplemental videos.

# 2. Supplemental Results

## 2.1. Data Acquisition

Population characteristics are summarized in 1.

## 2.2. Statistical Analysis

### *2.2.1. Linear Mixed-Effect Analysis*

The results of the linear mixed-effect analysis are summarized in Table 2. $\Delta SI$ was found to increase during breath-holds compared to BH start, with estimated changes of 2.39% for BH <10 s (95% CI: -0.55–5.33, p = 0.11), 3.35% for BH 10–20 s (0.45–6.26, p = 0.02), 4.89% for BH 20–30 s (1.80–7.98, p < 0.01), and 5.12% for BH >30 s (1.86–8.37, p < 0.01). However, $\Delta SI$ was highest during normal breathing (6.22%, 3.32-9.13, p<0.01). Sequence type (RT FLASH or RT bSSFP) and RR interval showed little evidence of an association with $\Delta SI$. The random effect variance was 2.52, indicating high inter-subject variability.

### *2.2.2. Intra- and Inter-sequence Comparisons*

Comparisons of breathing-interval pairs within each sequence using model-based marginal contrasts (Table 2a and Fig. 2b) showed a progressive increase in $\Delta SI$ throughout the breath-hold across all sequences. Although all sequences exhibited similar trends toward reduced $\Delta SI$ during hyperventilation compared with normal breathing, this decrease was most pronounced for the ECG-triggered bSSFP sequence. As the acquisition order was fixed and temporal drift may have contributed to the observed $\Delta SI$ changes, inter-sequence differences should be interpreted cautiously. Comparisons of sequence type pairs for selected breathing-intervals (Table 3) suggested that RT FLASH and RT bSSFP produced signal changes broadly comparable to the ECG-triggered bSSFP sequence. The largest discrepancies were observed during hyperventilation, where ECG-triggered bSSFP exhibited lower $\Delta SI$ than both RT FLASH and RT bSSFP. Bland-Altman analysis (Fig. 3) of global, end-systolic $\Delta SI$ during breath-hold periods showed that the ECG-triggered bSSFP–RT FLASH comparison exhibited a larger systematic bias (mean difference, 0.98) than the ECG-triggered bSSFP–RT bSSFP comparison (mean difference, -0.35), with relatively wide limits of agreement in both cases. As summarized in Table 4, which reports inter-sequence intraclass correlation coefficients (ICC, type 3k ) for ECG-triggered bSSFP versus RT FLASH comparisons per subject, the highest agreement was observed for subjects 5, 9, 8, and 10 (ICC 0.83–0.92), and the lowest for subjects 1, 4, 6, and 7 (ICC 0.47–0.68). However, the wide 95% confidence intervals indicate limited precision of these estimates. Across all subjects, the ICC was 0.65 [0.37, 0.81].

Oxygenation map heterogeneity was quantified as the mean gradient magnitude between neighboring pixels:

$$H = \frac{1}{T}\sum_{t=1}^{T}\frac{1}{M}\sum_{i,j}\sqrt{\left(\Delta SI_{i+1,j,t} - \Delta SI_{i,j,t}\right)^2 + \left(\Delta SI_{i,j+1,t} - \Delta SI_{i,j,t}\right)^2}$$

**Table 2**
Linear mixed-effects analysis was performed on $\Delta SI$, with sequence type, breathing interval, their interaction, and RR interval (ECG R-to-R interval, a proxy for heart rate) included as fixed effects, and subject-specific random intercepts. Estimates and confidence intervals represent changes in $\Delta SI(\%)$ relative to the respective reference category. The RR interval coefficient represents the estimated change in $\Delta SI(\%)$ per 1-second increase in RR interval. P-values are reported for descriptive purposes.

| **Effect** | **Estimate ($\Delta SI$, %)** | **95% CI ($\Delta SI$, %)** | **p-value** |
|---|---|---|---|
| Intercept | -0.31 | -6.07 – 5.46 | 0.92 |
| *Sequence (vs ECG-triggered bSSFP)* | | | |
| RT FLASH | -0.04 | -2.95 – 2.88 | 0.98 |
| RT bSSFP | -0.01 | -2.86 – 2.84 | 0.99 |
| *Interval (vs BH start)* | | | |
| Normal | **6.22** | 3.32 – 9.13 | **<0.01** |
| HV 30–40 s | 0.10 | -2.84 – 3.04 | 0.95 |
| HV 40–50 s | 0.98 | -1.95 – 3.91 | 0.51 |
| HV 50–60 s | 1.22 | -1.81 – 4.25 | 0.43 |
| BH <10 s | 2.39 | -0.55 – 5.33 | 0.11 |
| BH 10–20 s | **3.35** | 0.45 – 6.26 | **0.02** |
| BH 20–30 s | **4.89** | 1.80 – 7.98 | **<0.01** |
| BH >30 s | **5.12** | 1.86 – 8.37 | **<0.01** |
| *Sequence × Interval interactions (vs ECG-triggered bSSFP × BH start)* | | | |
| RT FLASH × Normal | -1.00 | -5.03 – 3.02 | 0.63 |
| RT bSSFP × Normal | 0.37 | -3.65 – 4.40 | 0.86 |
| RT FLASH × HV 30–40 s | 3.67 | -0.41 – 7.75 | 0.08 |
| RT bSSFP × HV 30–40 s | **4.33** | 0.25 – 8.41 | **0.04** |
| RT FLASH × HV 40–50 s | 3.61 | -0.47 – 7.70 | 0.08 |
| RT bSSFP × HV 40–50 s | 4.06 | -0.03 – 8.14 | 0.05 |
| RT FLASH × HV 50–60 s | 2.82 | -1.33 – 6.98 | 0.18 |
| RT bSSFP × HV 50–60 s | 2.28 | -1.88 – 6.43 | 0.28 |
| RT FLASH × BH <10 s | -0.83 | -4.91 – 3.25 | 0.69 |
| RT bSSFP × BH <10 s | -0.02 | -4.10 – 4.06 | 0.99 |
| RT FLASH × BH 10–20 s | -0.44 | -4.46 – 3.58 | 0.83 |
| RT bSSFP × BH 10–20 s | 0.62 | -3.41 – 4.64 | 0.77 |
| RT FLASH × BH 20–30 s | -1.27 | -5.36 – 2.81 | 0.54 |
| RT bSSFP × BH 20–30 s | 0.23 | -3.85 – 4.31 | 0.91 |
| RT FLASH × BH >30 s | -1.06 | -5.35 – 3.23 | 0.63 |
| RT bSSFP × BH >30 s | 0.25 | -3.99 – 4.50 | 0.91 |
| RR interval | 0.45 | -7.27 – 8.16 | 0.91 |
| *Random effect (subject variance) 2.52* | | | |

where $\Delta SI_{i,j,t}$ denotes the oxygenation map value at pixel $(i, j)$ and time frame $t$, $T$ the number of included time frames, and $M$ the number of myocardial pixels considered per frame. To avoid boundary effects, the myocardial mask was eroded by one pixel, and gradients were evaluated only within the eroded region. During the breath-hold period, the median [IQR] heterogeneity of end-systolic myocardial oxygenation maps was 5.9 [5.3, 6.6] for ECG-triggered bSSFP, 5.4 [4.0, 5.8] for RT bSSFP, and 2.7 [2.4, 2.9] for RT FLASH.

## 2.3. AHA Segment Analysis

AHA segment variations for end-systolic frames during breath-holding are summarized in Fig. 4. Considering cohort average statistics, the three sequences showed a similarly high average SI increase in the septal segments (8; 9), whereas larger discrepancies were observed in the anterior (7; 12) and inferior (10; 11) segments. As artifact distributions and segment rejection rates differed across sequences, the comparison of sequences may be biased (see Table 5). The RT FLASH sequence yielded reconstructions with least imaging artifacts, whereas the bSSFP sequences often demonstrated banding artifacts at the heart-lung interface. The presented artifact distributions also reflect regions that were excluded due to unreliable signal behavior arising from incorrect image registration. Inspection of the segment

| Effect | Estimate | 95% CI | p-value |
|---|---|---|---|
| *Normal vs HV 30–40 s* | | | |
| ECG-triggered bSSFP | **6.12** | 3.05 – 9.19 | **<0.01** |
| RT bSSFP | 2.16 | -0.89 – 5.21 | 0.16 |
| RT FLASH | 1.45 | -1.50 – 4.40 | 0.33 |
| *Normal vs HV 40–50 s* | | | |
| ECG-triggered bSSFP | **5.24** | 2.22 – 8.26 | **<0.01** |
| RT bSSFP | 1.56 | -1.45 – 4.56 | 0.31 |
| RT FLASH | 0.62 | -2.31 – 3.55 | 0.68 |
| *Normal vs HV 50–60 s* | | | |
| ECG-triggered bSSFP | **5.01** | 1.87 – 8.14 | **<0.01** |
| RT bSSFP | **3.10** | 0.07 – 6.14 | 0.05 |
| RT FLASH | 1.18 | -1.77 – 4.12 | 0.43 |
| *BH start vs BH <10 s* | | | |
| ECG-triggered bSSFP | -2.39 | -5.33 – 0.55 | 0.11 |
| RT bSSFP | -2.37 | -5.22 – 0.48 | 0.10 |
| RT FLASH | -1.56 | -4.41 – 1.29 | 0.28 |
| *BH start vs BH 10–20 s* | | | |
| ECG-triggered bSSFP | **-3.35** | -6.25 – -0.44 | **0.02** |
| RT bSSFP | **-3.96** | -6.86 – -1.07 | **<0.01** |
| RT FLASH | **-2.91** | -5.80 – -0.02 | 0.05 |
| *BH start vs BH 20–30 s* | | | |
| ECG-triggered bSSFP | **-4.89** | -7.98 – -1.79 | **<0.01** |
| RT bSSFP | **-5.12** | -8.09 – -2.15 | **<0.01** |
| RT FLASH | **-3.62** | -6.57 – -0.67 | **0.02** |
| *BH start vs BH >30 s* | | | |
| ECG-triggered bSSFP | **-5.12** | -8.37 – -1.86 | **<0.01** |
| RT bSSFP | **-5.37** | -8.40 – -2.34 | **<0.01** |
| RT FLASH | **-4.06** | -7.12 – -1.00 | **<0.01** |

(a)

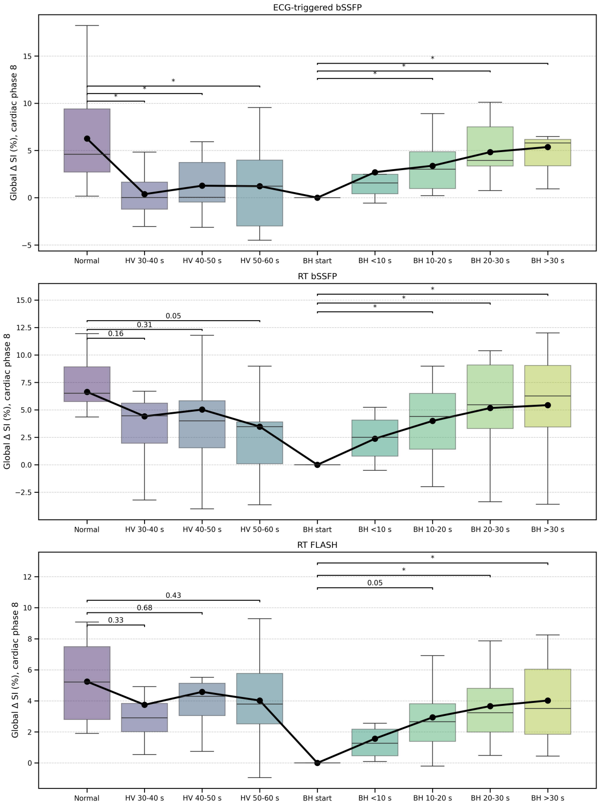


(b)

**Figure 2:** Intra-sequence $\Delta SI$ comparisons between breathing interval pairs, derived from model-based marginal contrasts of the linear mixed-effects model. (b) Boxplot of $\Delta SI$ across breathing intervals for each sequence, overlaid with the mean $\Delta SI$ per sequence and interval (solid lines). P-values are reported for descriptive purposes.

statistics for a selected breathing interval (BH 20–30 s) at the subject level (Fig. 5) revealed pronounced inter-subject and inter-sequence variability, suggesting limited reliability for segment-wise analysis.

## 2.4. Cardiac Cycle Analysis

When extending the $\Delta SI$ analysis from the end-systolic phase ($c = 8$) to the entire cardiac cycle, pronounced intra-sequence variations between systolic and diastolic frames were observed, as shown in Fig. 6-7. The highest global $\Delta SI$ values occurred during contracting cardiac phases. In contrast, diastolic frames (~12–19) of both RT sequences, contrary to expectation, exhibited lower $\Delta SI$ values at BH end than at BH start. It remains to be investigated whether the proposed pipeline is suitable for oxygenation assessment during diastole, or whether the thinner myocardial masks encountered in this phase are too susceptible to segmentation and registration inaccuracies. Comparing the cardiac-cycle curves for the global myocardium (left) and the septal region (right), the latter appeared to provide estimates more closely aligned with the expected behavior for the RT FLASH sequence. A recent study by Dresselaers et al. [8] demonstrates that through-plane motion velocity and spin magnetization history strongly influence signal intensity in 2D bSSFP cine imaging. The authors report cyclic myocardial signal intensity variations in mid-ventricular 2D bSSFP cine imaging, characterized by a three-peak pattern associated with contraction and relaxation phases during systole, early diastole, and end-diastole. Comparable motion-related signal intensity fluctuations observed in spoiled gradient echo simulations and phantom experiments indicate that these effects are not unique to balanced steady-state behavior. It therefore remains to be investigated to what extent through-plane motion may have contributed to the measured signal intensity differences in the present study.

**Table 3**
Inter-sequence $\Delta SI$ comparisons within each breathing interval, derived from model-based marginal contrasts of the linear mixed-effects model. P-values are reported for descriptive purposes.

| Effect | Estimate | 95% CI | p-value |
|---|---|---|---|
| *RT bSSFP vs ECG-triggered bSSFP* | | | |
| Normal | 0.36 | -2.50 – 3.22 | 0.81 |
| HV 30–40 s | 4.32 | 1.39 – 7.25 | **<0.01** |
| HV 40–50 s | 4.04 | 1.12 – 6.97 | **<0.01** |
| HV 50–60 s | 2.26 | -0.76 – 5.29 | 0.14 |
| BH start | -0.01 | -2.86 – 2.84 | 0.99 |
| BH <10 s | -0.03 | -2.96 – 2.89 | 0.98 |
| BH 10–20 s | 0.60 | -2.24 – 3.45 | 0.68 |
| BH 20–30 s | 0.22 | -2.70 – 3.15 | 0.88 |
| BH >30 s | 0.24 | -2.93 – 3.41 | 0.88 |
| *RT FLASH vs ECG-triggered bSSFP* | | | |
| Normal | -1.04 | -3.91 – 1.83 | 0.48 |
| HV 30–40 s | 3.63 | 0.65 – 6.61 | **0.02** |
| HV 40–50 s | 3.58 | 0.61 – 6.54 | **0.02** |
| HV 50–60 s | 2.79 | -0.27 – 5.85 | 0.07 |
| BH start | -0.04 | -2.95 – 2.88 | 0.98 |
| BH <10 s | -0.87 | -3.83 – 2.10 | 0.57 |
| BH 10–20 s | -0.48 | -3.38 – 2.42 | 0.75 |
| BH 20–30 s | -1.31 | -4.26 – 1.65 | 0.39 |
| BH >30 s | -1.09 | -4.38 – 2.19 | 0.51 |
| *RT bSSFP vs RT FLASH* | | | |
| Normal | 1.40 | -1.44 – 4.24 | 0.33 |
| HV 30–40 s | 0.69 | -2.18 – 3.56 | 0.64 |
| HV 40–50 s | 0.47 | -2.40 – 3.33 | 0.75 |
| HV 50–60 s | -0.52 | -3.39 – 2.35 | 0.72 |
| BH start | 0.03 | -2.85 – 2.90 | 0.99 |
| BH <10 s | 0.83 | -2.04 – 3.70 | 0.57 |
| BH 10–20 s | 1.08 | -1.80 – 3.96 | 0.46 |
| BH 20–30 s | 1.53 | -1.34 – 4.40 | 0.30 |
| BH >30 s | 1.34 | -1.60 – 4.28 | 0.37 |

## 2.5. Sensitivity Analysis

A sensitivity analysis was conducted to assess the impact of processing framework hyperparameter choices, including artifact rejection thresholds for excluding segments from the segment-wise analysis (33%), segmentation mask erosion/dilation (none), and temporal median filter window sizes (5 frames for the RT sequences and none for ECG-triggered bSSFP due to its lower temporal resolution). Artifact rejection thresholds were applied only at the segment level and not to the global myocardium. Morphological post-processing of the segmentation masks used a disk-shaped structuring element of size 1. Fig. 8 visualizes the sensitivity analysis results for global myocardium $\Delta SI$ at end-systole, focusing on the impact of median filter window size and segmentation mask post-processing. Larger filter window sizes resulted in reduced oxygenation response estimates. Filtering was performed within each cardiac phase sequence: for the RT acquisitions, a window size of 5 typically corresponded to temporal smoothing over several seconds depending on heart rate and temporal resolution, whereas for the ECG-triggered sequence it spanned substantially longer time intervals due to the lower temporal resolution. The effect of segmentation mask post-processing was less pronounced and less consistent across sequences. Mask dilation showed a tendency toward increased $\Delta SI$ for the ECG-triggered bSSFP sequence, a minor effect for RT bSSFP, and a tendency toward decreased $\Delta SI$ for the RT FLASH sequence. The interpretation of individual segments appeared unreliable for segments with a high prevalence of artifacts, leading to extensive segment exclusion and a strong impact on cohort-level statistics (Fig. 9).

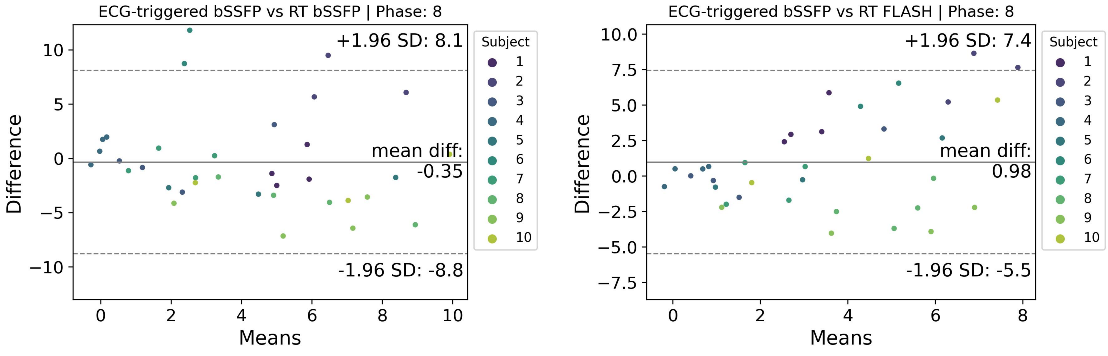


(a) (b)

**Figure 3:** Bland-Altman analysis comparing sequence types (including solely breath-hold interval samples) with subjects indicated by color.

| Volunteer | ICC | 95% CI |
|---|---|---|
| 1 | 0.47 | [-4.09, 0.94] |
| 2 | 0.71 | [-3.51, 0.98] |
| 3 | 0.78 | [-1.09, 0.98] |
| 4 | 0.59 | [-2.97, 0.96] |
| 5 | 0.92 | [-0.26, 0.99] |
| 6 | 0.62 | [-13.77, 0.99] |
| 7 | 0.68 | [-2.04, 0.97] |
| 8 | 0.89 | [-0.04, 0.99] |
| 9 | 0.92 | [0.27, 0.99] |
| 10 | 0.83 | [-1.58, 0.99] |

**Table 4**
Inter-sequence (ECG-triggered bSSFP vs RT FLASH) intraclass correlation coefficient for global end-systole $\Delta SI$ during breath-hold intervals.

| | Segment index | | | | | |
|---|---|---|---|---|---|---|
| Sequence | 7 | 8 | 9 | 10 | 11 | 12 |
| ECG-triggered bSSFP | 0 | 0 | 1 | 5 | 2 | 0 |
| RT bSSFP | 4 | 0 | 0 | 7 | 4 | 1 |
| RT FLASH | 2 | 0 | 0 | 1 | 0 | 0 |

**Table 5**
Artifact rejection counts for end-systolic frames per sequence and segment index.

## 2.6. Repeatability Analysis

### *2.6.1. Quality Control Statistics*

Manual interactions were required for R-peak review, segmentation mask corrections, artifact delineation, and quality control. Most manual segmentation mask corrections were necessary for the RT FLASH sequence, likely due to its differing image contrast compared to the M&Ms training dataset used for nnU-Net model training, suggesting that fine-tuning the model on FLASH acquisitions could reduce manual annotation efforts. Averaged over reference frames and subjects, Dice scores computed between the automated and corrected myocardium segmentation masks were 0.88 for both ECG-triggered bSSFP and RT bSSFP, and 0.69 for the RT FLASH sequence. Regarding artifact delineation, 27, 63, and 19 pixels were excluded on average (again considering reference frames and subjects) for ECG-triggered bSSFP, RT bSSFP, and RT FLASH, respectively. Table 5 summarizes the segment rejection counts for end-systolic frames, considered for the AHA segment analysis. Frame exclusion and inclusion counts per sequence and breathing maneuver interval are provided in Table 6.

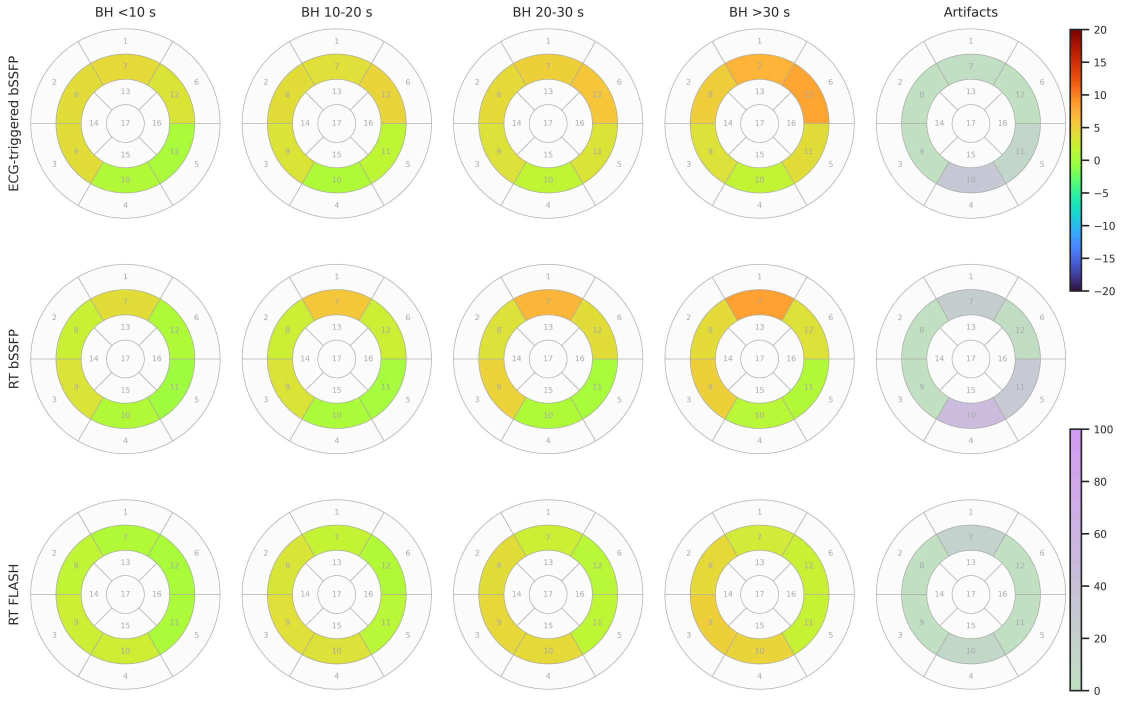


**Figure 4:** Average cohort segment-wise $\Delta SI(\%)$ statistics (end-systole; breath-holding) and artifact percentages.

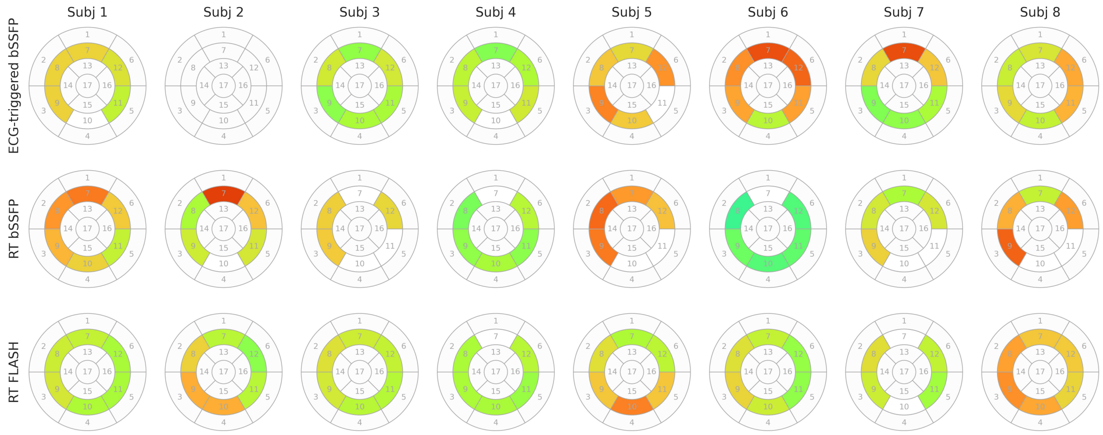


**Figure 5:** Per subject segment-wise $\Delta SI(\%)$ statistics for a selected breathing interval (end-systole; BH 20-30s).

### 2.6.2. *Repeated Reading*

Reproducibility was assessed by repeated readings performed by the same reader, including repeated manual contour correction and quality control, after an interval of approximately five months. The reader was blinded to previous annotations but not to the subject identifiers. The analysis was restricted to a subset of subjects (1–8) and to end-systolic frames ($c = 8$). Bland–Altman analysis indicated good agreement between the two readings for all sequences (Fig. 10).

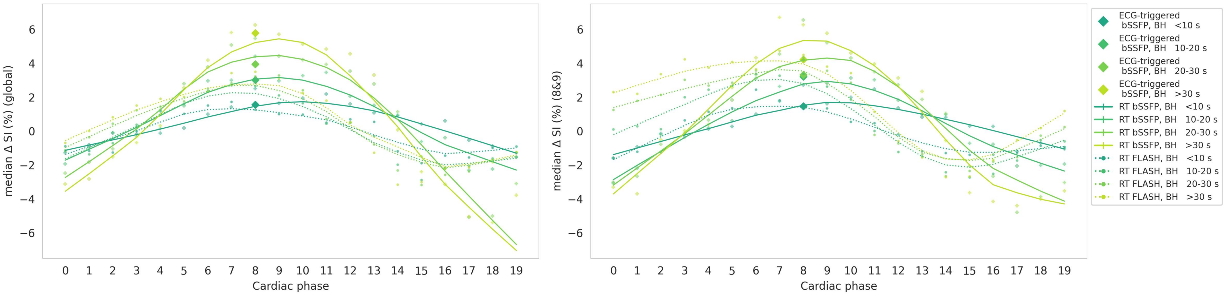


**Figure 6:** Median global (left) and septal (right) $\Delta SI(\%)$ oxygenation changes by breath-hold interval across sequences and cardiac phases, with curves fitted using locally estimated scatterplot smoothing (LOESS) with a smoothing span of 0.4. No curves were fitted for the ECG-triggered sequence, as it captured only a single cardiac phase.

### *2.6.3. Repeated Acquisition*

Repeatability was further assessed using repeated acquisitions of a single volunteer, who remained in the scanner for six breathing maneuvers. The following sequences were acquired in order: ECG-triggered bSSFP, RT bSSFP, RT FLASH, ECG-triggered bSSFP, RT bSSFP, and RT FLASH. In this section, the repeatability analysis was restricted to the RT FLASH sequence, as pronounced banding artifacts were observed in the RT bSSFP acquisitions and intermittent loss of the ECG signal occurred during the ECG-triggered bSSFP sequence. Figure 11a and 11b demonstrate similar breath-hold oxygenation responses and comparable RR-interval changes across the repeated acquisitions. The ICC for the RT FLASH sequence, with $\Delta SI$ assessed over 10s breathing intervals, was 0.90 (95% CI: 0.52-0.98).

## 2.7. Echo Time Ablations

To increase the $T_2^*$ contrast of the RT FLASH sequence, acquisitions were performed at four different echo times (1.61, 2.28, 4.28, and 7.28ms). Among these, TE = 2.28 ms was identified as the longest achievable echo time with acceptable imaging artifacts. As shown in Fig. 11c, this increase in echo time did not result in a notable increase in the breath-hold oxygenation response metrics. Compared with the first RT FLASH acquisition at TE = 1.61 ms, the ICC for the RT FLASH sequence, with $\Delta SI$ assessed over 10 s breathing intervals, was 0.86 (95% CI: 0.32-0.97).

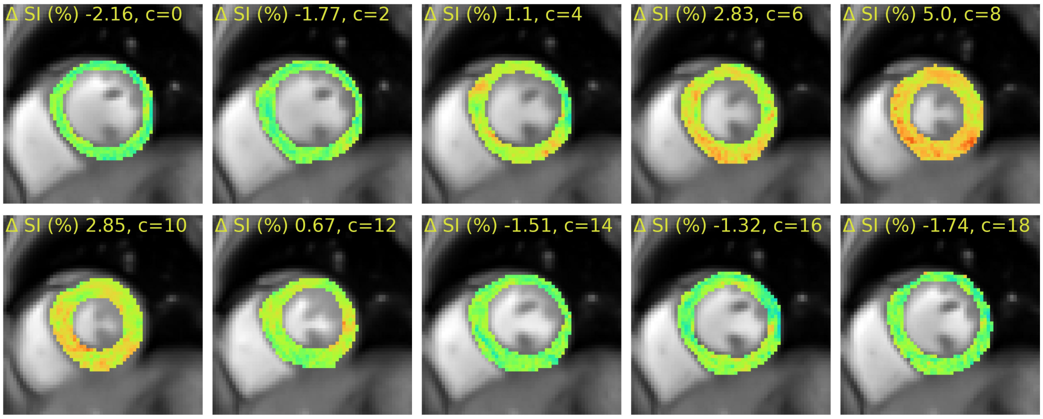


(a)

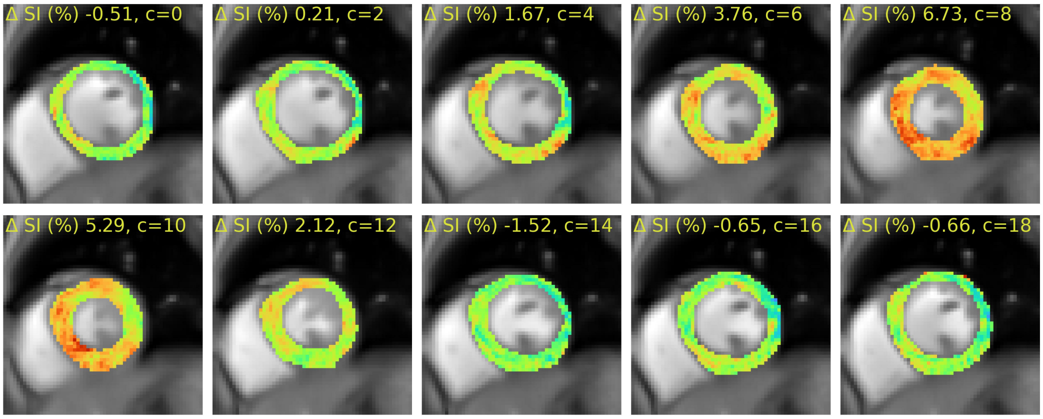


(b)

**Figure 7:** RT FLASH oxygenation maps demonstrating the cardiac cycle ($c \in [0, 2, ..., 18]$) $\Delta SI\%$ variations for volunteer 8. Averaged non-rigidly-registered images and SI maps were obtained for (a) BH start <10 s and (b) BH end 20-30s.

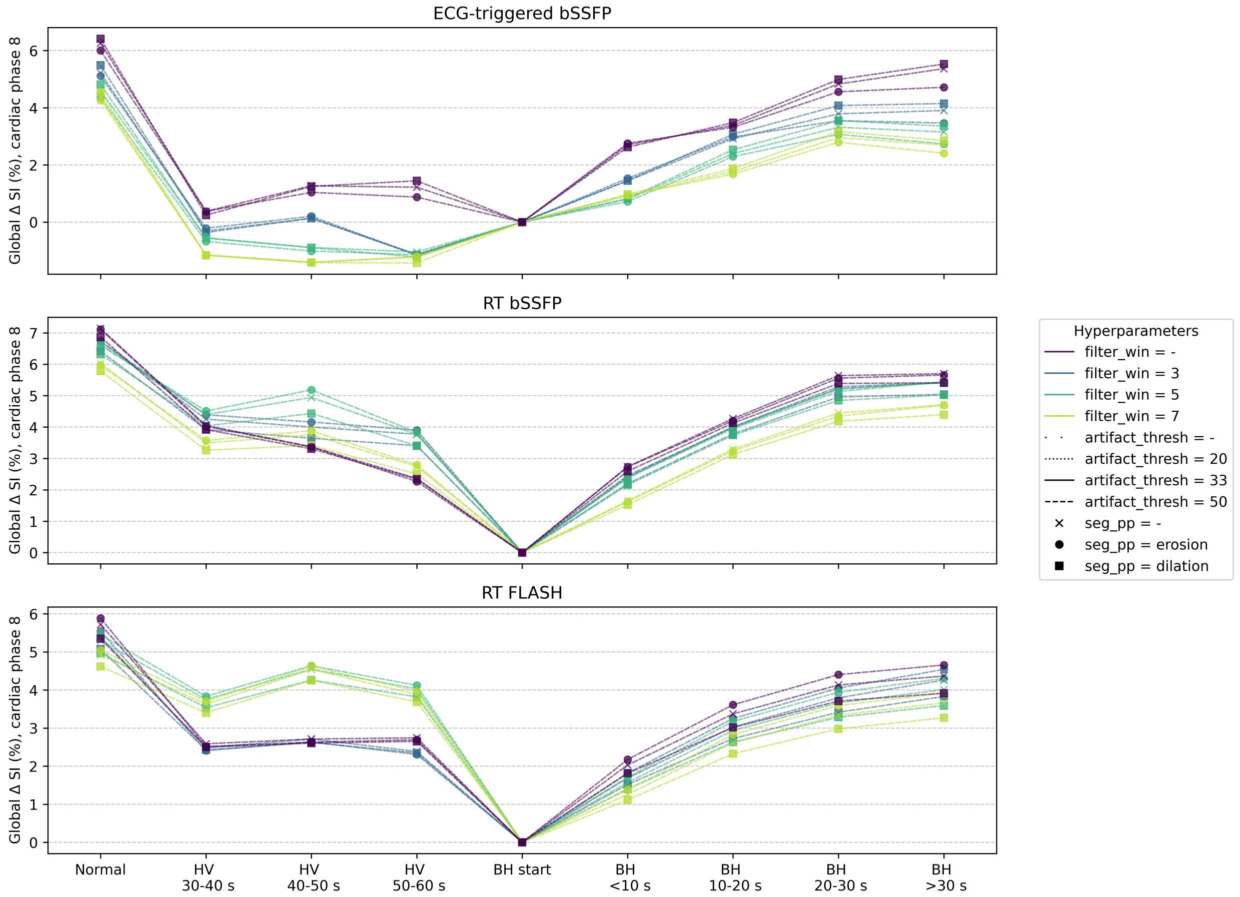


**Figure 8:** Sensitivity analysis for global myocardium $\Delta SI$ at end-systole for temporal median filter size (filter_win) and segmentation mask postprocessing (seg_pp), showing mean $\Delta SI$ per sequence and interval. Note that artifact thresholds were not applied to the global myocardium but only individual AHA segments.

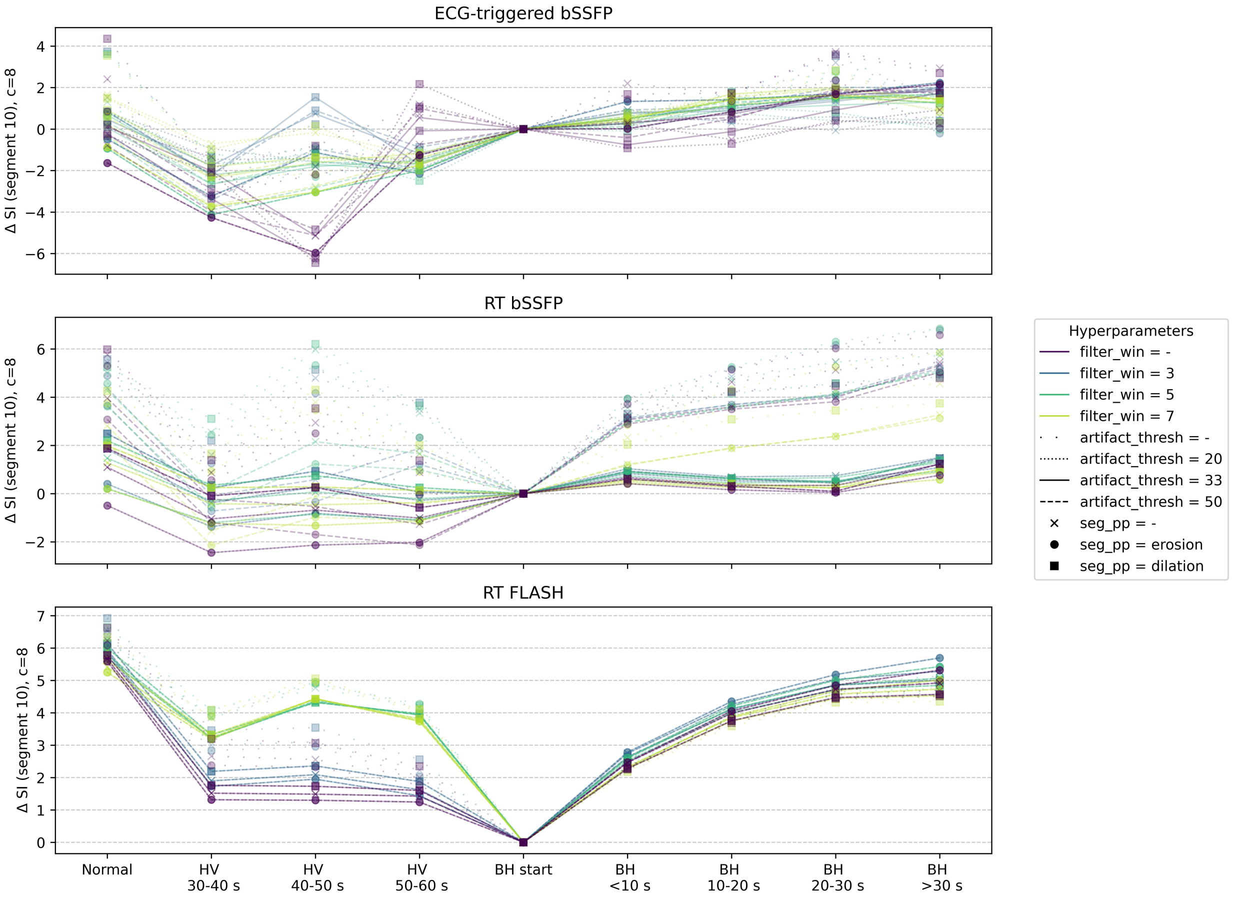


**Figure 9:** Sensitivity analysis for $\Delta SI$ of segment 10 at end-systole for temporal median filter size (filter_win) and segmentation mask postprocessing (seg_pp), showing mean $\Delta SI$ per sequence and interval.

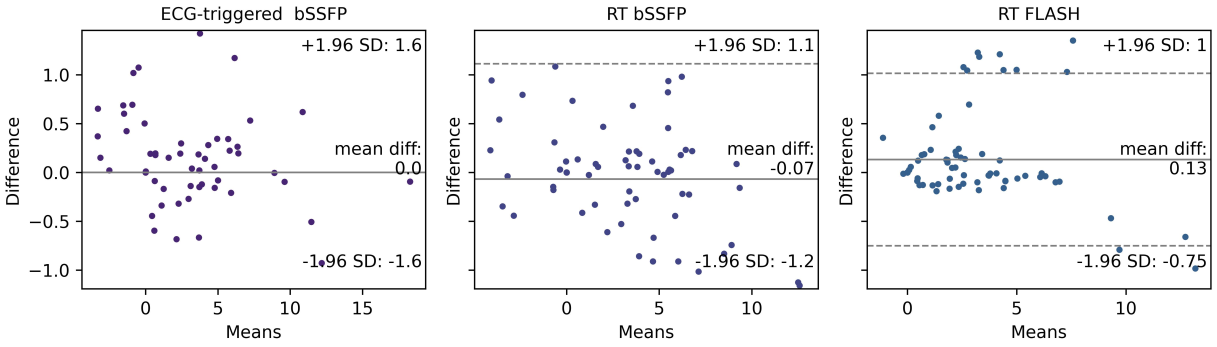


**Figure 10:** Bland-Altman analysis for $\Delta SI$ agreement obtained by repeated manual contouring correction and quality control of end-systolic frames.

| Sequence | Interval | Excluded (all cardiac phases) | | | | Included | |
|---|---|---|---|---|---|---|---|
| | | Resp. amplitude | Image quality | Synchronization | Registration | All cardiac phases | End-systole (c=8) |
| ECG-triggered bSSFP | Normal | 18 | 0 | 0 | 3 | 71 | 71 |
| | HV 30–40 s | 7 | 0 | 0 | 1 | 12 | 12 |
| | HV 40–50 s | 6 | 0 | 1 | 0 | 10 | 10 |
| | HV 50–60 s | 9 | 0 | 1 | 0 | 12 | 12 |
| | BH <10 s | 1 | 0 | 0 | 0 | 10 | 10 |
| | BH 10–20 s | 0 | 0 | 0 | 0 | 14 | 14 |
| | BH 20–30 s | 0 | 0 | 0 | 0 | 14 | 14 |
| | BH >30 s | 0 | 0 | 0 | 0 | 13 | 13 |
| RT bSSFP | Normal | 3391 | 0 | 0 | 0 | 12845 | 629 |
| | HV 30–40 s | 986 | 0 | 0 | 3 | 2015 | 105 |
| | HV 40–50 s | 1033 | 0 | 0 | 2 | 1969 | 112 |
| | HV 50–60 s | 1111 | 0 | 0 | 2 | 2561 | 133 |
| | BH <10 s | 0 | 0 | 0 | 0 | 2800 | 141 |
| | BH 10–20 s | 0 | 0 | 0 | 0 | 3007 | 150 |
| | BH 20–30 s | 0 | 0 | 24 | 0 | 2978 | 144 |
| | BH >30 s | 0 | 0 | 0 | 0 | 3166 | 149 |
| RT FLASH | Normal | 5193 | 0 | 0 | 6 | 21907 | 1082 |
| | HV 30–40 s | 1544 | 0 | 0 | 0 | 3226 | 164 |
| | HV 40–50 s | 1555 | 0 | 0 | 0 | 3211 | 165 |
| | HV 50–60 s | 1850 | 0 | 0 | 0 | 3893 | 210 |
| | BH <10 s | 0 | 0 | 0 | 0 | 4567 | 227 |
| | BH 10–20 s | 0 | 0 | 0 | 0 | 4770 | 225 |
| | BH 20–30 s | 0 | 0 | 0 | 0 | 4507 | 220 |
| | BH >30 s | 0 | 0 | 0 | 0 | 2147 | 98 |

Table 6: Quality control summary showing counts of excluded frames by reason and the resulting count of included frames (for all cardiac phases and for end-systolic frames), summed across all volunteers.

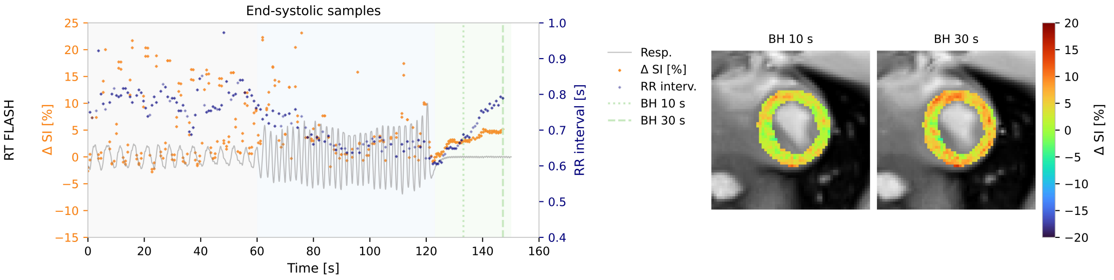


(a)

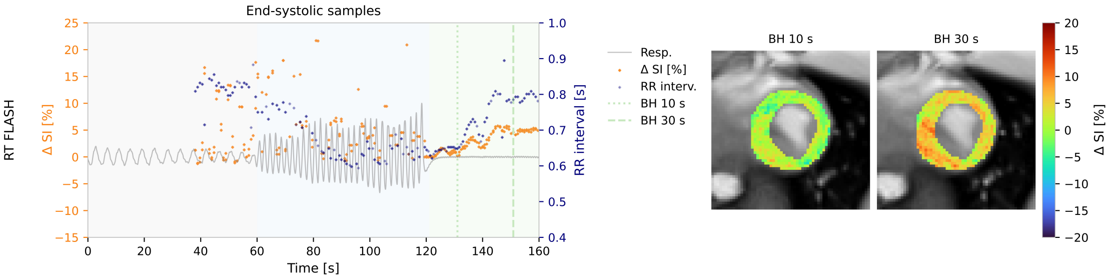


(b)

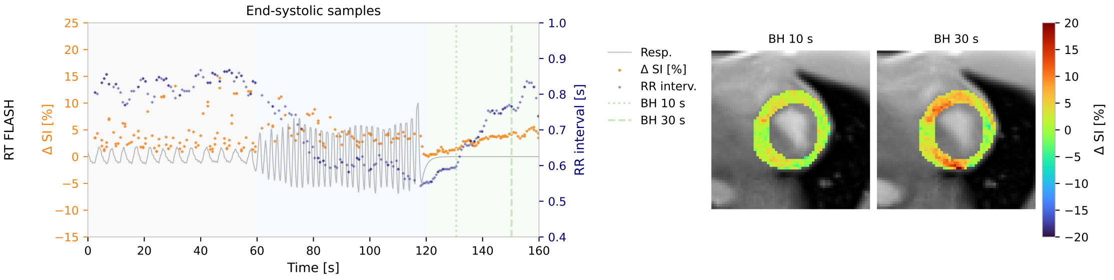


(c)

**Figure 11:** Same-day repeatability assessment for repeated RT FLASH acquistions (a) and (b), with parts of normal breathing interval excluded due to loss of the ECG signal. (c) repeated acquisition with higher echo time (TE=2.28). Left: Global $\Delta SI$ and RR interval throughout the breathing maneuver (gray = normal breathing, blue = hyperventilation, green = breath-hold) for, from top to bottom, ECG-triggered bSSFP, real-time bSSFP, and real-time FLASH. Y-axis labels and ticks are color-coded to match the respective data series (orange = $\Delta SI$, blue = RR interval). Right: Oxygenation maps at two breath-hold time points (samples closest to $10\,\mathrm{s}$ and $30\,\mathrm{s}$ of breath-hold), corresponding to the vertical dashed lines on the left. RR interval refers to the ECG R-to-R interval, a proxy for heart rate.

# References


[1] Martin Uecker, Shuo Zhang, Dirk Voit, Alexander Karaus, Klaus-Dietmar Merboldt, and Jens Frahm. Real-time MRI at a resolution of 20 ms. *NMR in Biomedicine*, 23(8):986–994, 2010.

[2] Jens Frahm, Sebastian Schätz, Markus Untenberger, Shuo Zhang, Dirk Voit, K Dietmar, Jan M Merboldt, Joachim Lotz, and Martin Uecker. On the temporal fidelity of nonlinear inverse reconstructions for real-time MRI–the motion challenge. *Open Medical Imaging Journal*, 8:1–7, 2014.

[3] Karyna Isaieva, Marc Fauvel, Nicolas Weber, Pierre-andré Vuissoz, Jacques Felblinger, Julien Oster, and Freddy Odille. A hardware and software system for MRI applications requiring external device data. *Magnetic Resonance in Medicine*, 88(3):1406–1418, 2022.

[4] Jeffrey A Feinstein, Frederick H Epstein, Andrew E Arai, Thomas KF Foo, Michael R Hartley, Robert S Balaban, and Steven D Wolff. Using cardiac phase to order reconstruction (CAPTOR): A method to improve diastolic images. *Journal of Magnetic Resonance Imaging*, 7(5): 794–798, 1997.

[5] Fabian Isensee, Paul F. Jaeger, Simon A. Kohl, Jens Petersen, and Klaus H. Maier-Hein. Pretrained nnU-Net model from the cMRI M&Ms challenge 2020. In *Statistical Atlases and Computational Models of the Heart: M&Ms and EMIDEC Challenges*, 2020. doi: 10.5281/zenodo.4134721. URL https://doi.org/10.5281/zenodo.4134721.

[6] Eleftherios Garyfallidis, Matthew Brett, Bagrat Amirbekian, Ariel Rokem, Stefan Van Der Walt, Maxime Descoteaux, Ian Nimmo-Smith, and Dipy Contributors. Dipy, a library for the analysis of diffusion MRI data. *Frontiers in neuroinformatics*, 8:8, 2014.

[7] MD Cerqueira, NJ Weissman, V Dilsizian, and AK Jacobs. Standardized myocardial segmentation and nomenclature for tomographic imaging of the heart: a statement for healthcare professionals from the cardiac imaging committee of the council on clinical cardiology of the american heart association. *Circulation*, 105(4):539–542, 2002.

[8] Tom Dresselaers, Frederik De Keyzer, Alexandru Cernicanu, Jan Bogaert, and Peter Gatehouse. Toward understanding the balanced steady-state free precession signal intensity changes in cine cardiac magnetic resonance imaging: A preliminary evaluation in healthy subjects pre-and postcontrast. *Journal of Cardiovascular Magnetic Resonance*, 27(2):101908, 2025.
